# Socioeconomic deprivation results in high rates of diabetic ketoacidosis at type 1 diabetes diagnosis in England: A multicentre observational study

**DOI:** 10.1111/dme.70102

**Published:** 2025-07-08

**Authors:** Chamila Balagamage, Chariklia Pieridou, Afiya Andrews, Prem Sundaram, Tabitha Randell, Fiona Campbell, James Young, Ruben H. Willemsen, Astha Soni, Meghan McGrath, M. Loredana Marcovecchio, Nisha Pargass, Dhaara Iyer, Renuka P. Dias

**Affiliations:** ^1^ Birmingham Women's and Children's NHS Foundation Trust Birmingham UK; ^2^ University Hospitals of Leicester NHS Trust Leicester UK; ^3^ Nottingham Children's Hospital Nottingham University Hospitals NHS Trust Nottingham UK; ^4^ Leeds Teaching Hospitals NHS Trust Leeds UK; ^5^ The Royal London Children's Hospital, Barts Health NHS Trust London UK; ^6^ Sheffield Children's NHS Foundation Trust Sheffield UK; ^7^ School of Clinical Medicine University of Cambridge Cambridge UK; ^8^ Department of Paediatrics University of Cambridge and Cambridge University Hospitals NHS Foundation Trust Cambridge UK; ^9^ The Royal Wolverhampton NHS Trust Wolverhampton UK; ^10^ University Hospitals Birmingham NHS Foundation Trust Heartlands UK; ^11^ Department of Applied Health Sciences University of Birmingham Birmingham UK

To the Editor,

1

The global incidence of type 1 diabetes continues to rise, yet a considerable proportion of children continue to present in diabetic ketoacidosis (DKA) at diagnosis despite improved recognition, education, and management strategies.^1^, ^2^ DKA remains a significant contributor to morbidity, long‐term glycaemic instability, and elevated healthcare costs.^3^, ^4^

Although international estimates of DKA incidence at diagnosis vary considerably, ranging from 13% to 80% in those under 20 years,^2^ the most recent UK data from the National Paediatric Diabetes Audit (NPDA) reported a 23.3% incidence of DKA at type 1 diabetes diagnosis in 2023.^5^ The reasons for this geographical variation are multifactorial and include delayed diagnosis due to non‐specific symptoms, particularly in younger children, as well as disparities related to socioeconomic deprivation and ethnicity.^1^, ^2^, ^3^ However, UK‐based data exploring these associations in large, diverse cohorts are limited.

We conducted a retrospective, multicentre observational study to investigate the sociodemographic factors associated with DKA at the time of type 1 diabetes diagnosis in children and adolescents. Data were collected from nine paediatric diabetes units across five English regions: West Midlands, East Midlands, Yorkshire and Humber, London and the South East, and East of England. All individuals younger than 18 years diagnosed with stage 3 type 1 diabetes between January 2023 and August 2024 were included. Key variables included ethnicity (self‐identified into 5 NHS‐defined categories), socioeconomic status (determined using the 2019 Index of Multiple Deprivation [IMD] quintiles based on postcode), and interpreter requirement.

DKA severity was defined using the British Society for Paediatric Endocrinology and Diabetes (BSPED) criteria.^6^ Of 539 eligible patients, 499 had complete data for full analysis (see Table 1). Overall, 45.5% presented in DKA, a rate substantially higher than national figures. The cohort was notably diverse (38.3% non‐white) and socioeconomically deprived (54.9% from IMD quintiles 1 and 2). There was regional variation in deprivation (lowest seen in the East of England: 2.6% in IMD1 vs. the West Midlands: 51.2%).

**TABLE 1:** Characteristics of cohort presenting with and without diabetic ketoacidosis at diagnosis of type 1 Diabetes.

Cohort characteristic	Entire cohort (*n* = 539) (%)	No DKA (*n* = 272) (%)	DKA (*n* = 227) (%)	No DKA versus DKA, *p*‐value
Age at diagnosis (*n* = 539)	10.0 [7–13]	10.0 [7–13.55]	10.3 [7–13]	0.27
Gender (*n* = 539)	Male: 293/539 (54.4) Female: 246/539 (45.6)	Male 149 (54.8) Female 123 (45.2)	Male 125 (55.1) Female 102 (44.9)	0.38
Ethnicity (*n* = 539)	White 336 (62.3) Non‐white 203 (37.7) Asian 79 (14.7) Black 51 (9.5) Mixed 24 (4.4) Other ethnicities 49 (9.1)	White: 175 (64.7) Non‐white: 97 (35.3) Asian: 42 (15.4) Black: 22 (8) Mixed: 15 (5.5) Other ethnicities: 18 (6.6)	White: 134 (59) Non‐white: 93 (41) Asian: 33 (14.5) Black: 26 (11.5) Mixed: 8 (3.5) Other ethnicities: 26 (11.5)	0.17
Index of multiple deprivation quintiles (*n* = 525)	Quintile 1: 172 (32.8) Quintile 2: 116 (22.1) Quintile 3: 76 (14.5) Quintile 4: 79 (15) Quintile 5: 82 (15.6)	Quintile 1: 80 (29.4) Quintile 2: 51 (18.8) Quintile 3: 43 (15.8) Quintile 4: 44 (16.2) Quintile 5: 54 (19.9)	Quintile 1: 86 (37.9) Quintile 2: 61 (26.9) Quintile 3: 28 (12.3) Quintile 4: 31 (13.7) Quintile 5: 21 (9.3)	0.0013*
Interpreter status (*n* = 535)	Interpreter 36 (6.7) No Interpreter 501 (93.3)	Interpreter 17 (6.2) No Interpreter 255 (93.8)	Interpreter 16 (7) No Interpreter 210 (92.5)	0.72
Family history of T1D (*n* = 486)	Positive FH 152 (30.9) No FH 340 (69.1)	Positive FH 89 (32.7) No FH 170 (62.5) Not Available: 13 (4.8)	Positive FH 52 (22.9) No FH 148 (65.2) Not Available: 27 (11.2)	0.07
Antibody status (*n* = 471)	Positive 422 (88.7) Negative 54 (11.3)	Positive 218 (87.2) Negative 32 (12.8)	Positive 178 (78.4.6) Negative 16 (7)	0.17
AAB positivity at diagnosis (*n* = 247)		No AAB: 22 (15.5) One AAB: 30 (21.2) Two AAB: 54 (38.0) ≥Three AAB: 36 (25.4)	No AAB: 6 (5.7) One AAB: 41 (39.0) Two AAB: 36 (34.3) ≥Three AAB: 22 (21.0)	
Diabetes severity (*n* = 506)	No DKA: 280 (54.6) Mild‐ Moderate DKA: 127 (24.7) Severe DKA: 106 (20.6)			

*Note*: Data are median [interquartile range] or *n* (%).

Abbreviations: AAB, autoantibody; FH, family history; DKA, diabetes ketoacidosis; T1D, ype 1 diabetes.

* *p*‐value <0.05 denotes statistical significance.

## 2 KEY FINDINGS

### 2.1 High incidence of DKA at diagnosis across all sites

The overall DKA rate in this cohort was 45.5%, significantly higher than the 23% reported in national audits.^5^ This discrepancy may be explained by the demographic characteristics of our study population. Most units participating in the study serve populations with disproportionately high levels of deprivation, and many regions lack public awareness campaigns in contrast to those successfully implemented in countries like Sweden or Australia, where DKA rates at diagnosis are much lower.^7^, ^8^

### 2.2 Socioeconomic deprivation is the strongest predictor of DKA presentation

Our analysis found a significant association between lower socioeconomic status and DKA at diagnosis. Children in the most deprived quintile (IMD1) had a 51.8% DKA rate compared to 28.0% in the least deprived quintile (IMD5, *p* = 0.0007). These findings align with established research showing that lower SES is associated with reduced access to care, delayed diagnosis, and lower health literacy.^9^ Furthermore, unit‐level analysis confirmed that centres serving more deprived communities had markedly higher DKA rates compared to units serving more affluent populations.

### 2.3 Ethnicity, age, and family history were not significant risk factors in this cohort

Although previous literature has suggested that ethnic minorities or younger children may present more frequently in DKA due to delayed recognition and healthcare disparities, our analysis did not find ethnicity or age to be a significant predictor of DKA presentation (*p* = 0.23 and *p* = 0.65, respectively). Similarly, having a family history of T1D did not confer a protective effect, which contrasts with other reports suggesting improved recognition of early symptoms in such families.^10^ One possible explanation may be that health literacy, rather than ethnicity or family experience alone, plays a more influential role in timely diagnosis.

### 2.4 Low interpreter use and universal NHS access may mitigate some barriers

Interpreter use was low (6.7%) across all centres and showed no significant difference between DKA and non‐DKA groups, suggesting that language barriers may not be a major driver of late presentation in this setting. This highlights the potential levelling effect of free universal healthcare, although disparities persist most notably through deprivation rather than cultural or linguistic access.

## 3 CONCLUSION

This study is the largest contemporary analysis of DKA presentation at type 1 diabetes diagnosis in England, which identified socioeconomic deprivation as the most prominent risk factor for this acute presentation. While ethnicity, language, and family history did not significantly influence outcomes in our cohort, the persistent high rates of DKA, particularly among deprived communities, point to a broader public health issue. Efforts to reduce DKA incidence must focus on improving public and professional awareness, especially in high‐risk populations.

We advocate for long‐term, multi‐stage awareness campaigns targeting deprived areas, alongside consideration of targeted islet autoantibody screening to support earlier diagnosis and education to prevent DKA. Addressing socioeconomic inequalities and improving health literacy are essential steps to reverse the growing trend of DKA at type 1 diabetes diagnosis in the UK.

## FUNDING INFORMATION

No funding has been received for this work. RPD is supported by the NIHR (Ref NIHR304587).

## CONFLICT OF INTEREST STATEMENT

RPD, MLM, FC, and RHW have received honoraria from Sanofi (participation in the advisory board for teplizumab). RHW has received consultancy fees from Sanofi (review of HCP and patient materials for teplizumab), conference attendance support from Sanofi, and speaker fees from Insulet; TR has received honoraria (participation in advisory boards in screening for type 1 diabetes) and lecture fees from Sanofi. FC has received speaker fees from Insulet. Other authors declared no conflict of interest.

## ETHICS APPROVAL

Institutional review board approval for clinical data review was obtained from Birmingham Women's and Children's (BWC) NHS Foundation Trust (reference: CARMS‐31593). All data collected from other sites were part of anonymised National Paediatric Audit data collection and submission, and therefore, specific additional ethical approval was not required.

## Data Availability

The data that support the findings of this study are available from the corresponding author upon reasonable request.
